# Deciphering the Role of microRNAs in BRD4-NUT Fusion Gene Induced NUT Midline Carcinoma

**DOI:** 10.6026/97320630013209

**Published:** 2017-06-30

**Authors:** Ekta Pathak, Bhavya, Divya Mishra, Neelam Atri, Rajeev Mishra

**Affiliations:** 1 Bioinformatics Department, MMV, Banaras Hindu University, India; 2 Botany Section, MMV, Banaras Hindu University, India

**Keywords:** microRNAs, BRD4-NUT, Fusion Gene, NUT midline carcinoma, bioinformatics approach

## Abstract

NUT midline carcinoma (NMC) is a very aggressive and lethal type of squamous epithelial cell cancer caused due to fusion of BRD4 and NUT genes. The gene fusion results into a new fusion protein that promotes oncogenesis. The detailed molecular mechanisms underlying the NMC are still not clear and new findings are urgently required to complement the current efforts. Abnormal microRNAs (miRNA) expression promotes tumour formation by modulating the functional expression of critical genes other than the parent genes involved in tumour cell proliferation or survival. Here, using Insilco methods, miRNA targeting the transcripts of parent genes (BRD4 and NUT) and the BRD4-NUT fusion gene were predicted. We investigated a situation, wherein abnormal miRNA expression in malignant cells could arise due to deletion and fusion of genomic regions encompassing the target site of miRNA genes. A set of 48 dysregulated miRNAs targeting the critical genes other than the parent genes (BRD4 and NUT) was identified. Functional enrichment analysis of KEGG pathways of target genes of these Ex-miRNAs implicates their role in cancer pathways. Amplification in the expression level of these miRNAs can be used for NMC diagnosis and prognosis.

## Background

NUT midline carcinoma (NMC) is a fatal form of undifferentiated epithelial cancer affecting both children and adults [1]. Commonly, the sites of occurrence of this cancer are head, neck and space in the thoracic cavity between the lungs. In the majority of cases, NMC is due to the fusion of the testis-specific nuclear gene NUT of chromosome 15 and the bromodomaincontaining gene BRD4 (bromodomain protein family member 4) on chromosome 19. The gene fusion results into a new fusion protein that markedly disrupts squamous cell differentiation and promotes oncogenesis [2]. The detailed molecular mechanisms underlying the NMC are still not clear and new findings are urgently required to complement the current efforts.

MicroRNAs (miRNAs) play a critical role in regulating target genes and are involved in the initiation and progression of cancer [3]. miRNAs are small, about 21 nucleotides long non-coding RNAs which usually target the mRNAs at their 3׳UTR region and inhibit their expression [4]. It has also been shown that miRNAs also target the CDS and 5׳UTR region of the mRNAs [5]. miRNAs are not very specific for their targets and a single miRNA can modulate the expression levels of several hundred to thousands of different mRNAs [3]. Only a small portion of the miRNAs, i.e., the about seven nucleotides long seed region, should be complementary to the mRNA sequence to bind with it and inhibit its expression [6]. In recent years, miRNA profiling and deep sequencing provided direct evidences of dysregulated miRNAs that target key genes like oncogenes or tumour repressor genes to induce tumour development. These miRNAs signature could be used to distinguish between tumoral and normal cells, and in some instances prognosis and the progression of cancer [7].

Here, we have idetified the miRNA targeting the transcripts of parent genes (BRD4 and NUT) and the BRD4-NUT fusion gene. We hypothesize that some of the miRNAs regulating their respective BRD4 and NUT gene expression may not be required after the BRD4-NUT gene fusion, as the regions targeted by these miRNAs are deleted. This may lead to an apparent amplification in the miRNAs, which in turn may regulate the pathways leading to cancer. A comparative study of miRNAs regulating fused gene and its parent gene was performed and their target genes were discovered. A functional enrichment analysis of KEGG pathway of target genes of these Ex-miRNAs shows its role in cancer pathways.

## Methodology

BRD4 gene (Refseq: NM_058243.2), NUT gene (GenBank: AF482429.1) and BRD4-NUT fusion gene (GenBank: AY166680.1) transcript sequences were retrieved from the NCBI Nucleotide database. In order to locate the deleted regions in the fusion gene transcript, the mRNA sequences of parent (BRD4 and NUT) were compared against the fusion gene (BRD4-NUT) using align two sequences option of NCBI- BlastN tool. Prediction of miRNAs targeting the BRD4, NUT and BRD4-NUT fusion gene transcripts was performed using MIRDB (http://mirdb.org/miRDB) [8]. For each miRNAs, miRWalk (http://www.umm.uniheidelberg. de/apps/zmf/mirwalk/) was used to predict miRNA targets and analyse plausible KEGG pathway enrichment of dysregulated miRNA targets. miRWalk dataset hosts experimentally verified miRNA-mRNA interactions as well as the information on the published pathway targets from the KEGG (http://www.genome.jp/kegg/)[9]. Since miRNAs can target 3׳UTR, CDS and 5׳UTR region of the mRNAs [5], therefore parameter for target search was set to include 3׳UTR, 5׳UTR and CDS regions of transcript. Minimum seed length was chosen as seven with p-value equal to 0.05.

## Results and Discussion

It is generally accepted that fusion genes are translated into fusion proteins, which are drivers of cancer pathway activation. On the other hand, it is well established that alterations in miRNA expression promote tumour formation. The dysregulated miRNAs modulate the functional expression of critical genes other than the parent genes involved in tumour cell proliferation or survival [3]. Recent findings suggested that abnormal miRNA expression in malignant cells could arise due to amplification or deletion of specific genomic regions encompassing miRNA genes [10]. Here, we investigated miRNAs targeting the parent BRD4 and NUT genes and BRD4- NUT fusion transcript. We assume that in case of BRD4-NUT gene fusion, transcripts of parent gene will no more available to be targeted by their corresponding miRNAs termed as “ExmiRNA”. This will lead to a situation where an apparent amplification and dysregulation by these miRNAs are possible.

**Figure 1:**
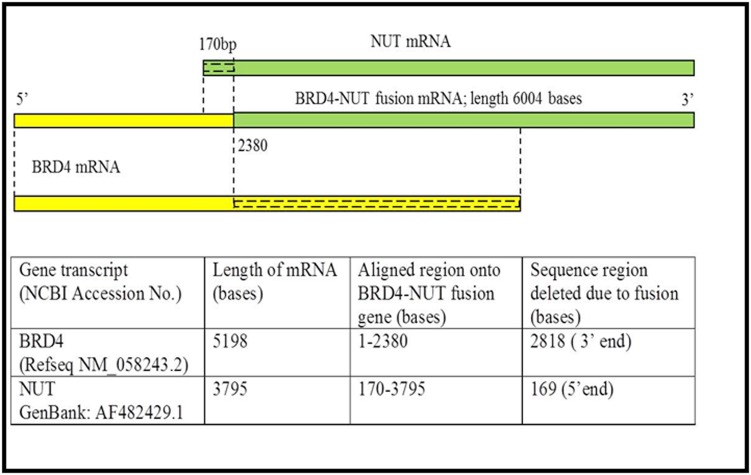
Schematic representation showing the alignment of BRD4 (yellow) and NUT (green) transcripts onto BRD4-NUT fused transcript. Deleted regions during the gene fusion event are marked with dashed line.

We predicted and compared the miRNA targeting the BRD4 and NUT transcripts region before and after the gene fusion events. Sequence alignment of parent BRD4, NUT and fusion BRD4-NUT mRNA sequences, using NCBI Blast tool, revealed that 2818 bases (54%) from 3׳ region of BRD4 and 170 bases from 5׳ region of NUT were deleted in the BRD4-NUT gene fusion transcript (Figure 1). miRNAs binding sites were identified in parent BRD4 and NUT transcripts using miRDB tool (see methods). 34, 33 and 19 miRNAs were predicted to target BRD4 mRNA, NUT mRNA and fused BRD4-NUT mRNA, respectively. Except, Has-miR-5193 targeting the fusion gene transcript, 18 miRNAs were common to target the parent BRD4 or NUT and fused BRD4-NUT transcript (Table 1). As evident in Figure 1, due to fusion transcript formation, the deleted regions will no more be available as targets for their respective miRNAs. Therefore, we compared the predicted miRNAs before and after the gene fusion and listed 48 such Ex-miRNAs (Table 2). We speculate that many of the these Ex-miRNA will be apparently amplified in cancer cells, as they are not required by their respective BRD4 and NUT targets in fusion gene condition, and hence show dysregulation in cancer.

In order to predict the Ex-miRNA, BRD4 (1-2830 region) and NUT (170-3795 region) of fused gene were subjected to miRNA prediction using miRDB. Out of 34 miRNAs of BRD4, 28 miRNA were no more required to target the same gene, when compared to its fused form. This situation arises because of the deletion of 2818 bases from 3׳end of BRD4 (Table 2). Similarly, out of 33 predicted miRNAs, 20 miRNA were predicted not to target NUT transcript compared to its fused form. The deletion of 170 bases from 5׳end of NUT justifies such situation. Overall, we have identified a list of 48 dysregulated “Ex-miRNAs” that target critical genes other than the parent genes. Functional enrichment analysis of KEGG pathway using miRWalk 2.0 tool revealed that Ex-miRNAs targeting 3׳UTR region of the target genes belongs to Pathways in cancer (Table 2). Also, miRNAs targeting the genes on their promoter, 5׳UTR, CDS and 3׳UTR regions were shown to be enriched for “Pathways in cancer” (Table 2). We suggest that an apparent amplification in Ex- microRNA can be used as molecular markers to diagnose NMC.

## Conclusion

A set of 18, 33, and 34 miRNAs were predicted to target the BRD4-NUT fusion gene transcript, BRD4 and NUT mRNA respectively. Comparative analysis of these miRNAs and functional enrichment of pathway revealed that a set of 48 miRNA might be dysregulated to target the critical genes other than the parent genes (BRD4 and NUT), causing the cancer. Amplification in the expression level of these miRNAs can be used for NMC diagnosis and prognosis.

## Figures and Tables

**Table 1:** List of miRNA targeting BRD4, NUT and BRD4-NUT fusion gene transcripts

miRNA targeting BRD4	miRNA targeting NUT	miRNA targeting BRD4-NUT
hsa-miR-4447	hsa-miR-6861-3p	hsa-miR-4279
hsa-miR-7159-5p	hsa-miR-1291	hsa-miR-4441
hsa-miR-3065-5p	hsa-miR-4306	hsa-miR-663b
hsa-miR-765	hsa-miR-4713-3p	hsa-miR-4303
hsa-miR-608	hsa-miR-4535	hsa-miR-4483
hsa-miR-4492	hsa-miR-6728-5p	hsa-miR-6861-3p
hsa-miR-4516	hsa-miR-3202	hsa-miR-1291
hsa-miR-4731-5p	hsa-miR-1285-3p	hsa-miR-4267
hsa-miR-4651	hsa-miR-4483	hsa-miR-4535
hsa-miR-4483	hsa-miR-6722-3p	hsa-miR-6728-5p
hsa-miR-6870-5p	hsa-miR-1909-3p	hsa-miR-4731-5p
hsa-miR-4272	hsa-miR-4441	hsa-miR-5193
hsa-miR-663b	hsa-miR-654-5p	hsa-miR-1285-3p
hsa-miR-4279	hsa-miR-541-3p	hsa-miR-654-5p
hsa-miR-5787	hsa-miR-4443	hsa-miR-541-3p
hsa-miR-185-3p	hsa-miR-330-5p	hsa-miR-6751-3p
hsa-miR-4472	hsa-miR-326	hsa-miR-1199-5p
hsa-miR-637	hsa-miR-6751-3p	hsa-miR-6764-5p
hsa-miR-1277-5p	hsa-miR-1199-5p	hsa-miR-1915-3p
hsa-miR-4784	hsa-miR-660-3p	
hsa-miR-3150b-3p	hsa-miR-7106-3p	
hsa-miR-92a-2-5p	hsa-miR-6745	
hsa-miR-6812-5p	hsa-miR-363-5p	
hsa-miR-149-3p	hsa-miR-4456	
hsa-miR-5703	hsa-miR-1231	
hsa-miR-4434	hsa-miR-6884-5p	
hsa-miR-4303	hsa-miR-3144-5p	
hsa-miR-4267	hsa-miR-5196-5p	
hsa-miR-2861	hsa-miR-4747-5p	
hsa-miR-4266	hsa-miR-328-3p	
hsa-miR-6819-5p	hsa-miR-6764-5p	
hsa-miR-6737-5p	hsa-miR-1915-3p	
hsa-miR-1275	hsa-miR-3123	
hsa-miR-4505		

**Table 2:** miRNA targeting the critical genes other than the parent genes show functional enrichment in cancer pathways.

Sl. No.	miRNAs	No. of gene targets implicated in cancer pathways
1.	hsa-miR-1231	235
2.	hsa-miR-1275	255
3.	hsa-miR-1277-5p	183
4.	has-miR-149-3p	270
5.	hsa-miR-185-3p	265
6.	hsa-miR-1909-3p	245
7.	hsa-miR-2861	237
8.	hsa-miR-3065-5p	192
9.	hsa-miR-3123	182
10.	hsa-miR-3144-5p	181
11.	hsa-miR-3150b-3p	238
12.	hsa-miR-3202	241
13.	hsa-miR-326	196
14.	hsa-miR-328-3p	187
15.	hsa-miR-330-5p	238
16.	hsa-miR-363-5p	213
17.	hsa-miR-4266	172
18.	hsa-miR-4272	166
19.	hsa-miR-4306	216
20.	hsa-miR-4434	194
21.	hsa-miR-4443	70
22.	hsa-miR-4447	83
23.	hsa-miR-4456	148
24.	hsa-miR-4472	148
25.	hsa-miR-4492	159
26.	hsa-miR-4505	201
27.	hsa-miR-4516	159
28.	hsa-miR-4651	100
29.	hsa-miR-4713-3p	188
30.	hsa-miR-4747-5p	194
31.	hsa-miR-4784	188
32.	hsa-miR-5196-5p	188
33.	hsa-miR-5703	179
34.	hsa-miR-5787	188
35.	hsa-miR-606	188
36.	hsa-miR-637	117
37.	hsa-miR-660-3p	105
38.	hsa-miR-6722-3p	85
39.	hsa-miR-6737-5p	188
40.	hsa-miR-6745	93
41.	hsa-miR-6812-5p	188
42.	hsa-miR-6819-5p	150
43.	hsa-miR-6870-5p	176
44.	hsa-miR-6884-5p	188
45.	hsa-miR-7106-3p	188
46.	hsa-miR-7159-5p	137
47.	hsa-miR-765	188
48.	hsa-miR-92a-2-5p	116

## References

[1] French C. (2014). Nat Rev Cancer.

[2] French CA. (2012). Annu Rev Pathol.

[3] Farazi TA (2013). Adv Exp Med Biol..

[4] Bartel D. (2004). Cell..

[5] Brodersen P,, Voinnet O, (2009). Nature reviews Molecular cell biology.

[6] Carthew RW, Sontheimer, EJ, (2009). Cell.

[7] Garzon R (2009). Blood..

[8] Wang X. (2008). Rna.

[9] Dweep H (2011). Journal of biomedical informatics.

[10] Iorio MV, Croce CM  (2017). EMBO Mol Med..

